# Top-Down Characterization of the Post-Translationally Modified Intact Periplasmic Proteome from the Bacterium *Novosphingobium aromaticivorans*

**DOI:** 10.1155/2013/279590

**Published:** 2013-03-10

**Authors:** Si Wu, Roslyn N. Brown, Samuel H. Payne, Da Meng, Rui Zhao, Nikola Tolić, Li Cao, Anil Shukla, Matthew E. Monroe, Ronald J. Moore, Mary S. Lipton, Ljiljana Paša-Tolić

**Affiliations:** ^1^Environmental Molecular Science Laboratory, Pacific Northwest National Laboratory, P.O. Box 999/MS K8-98, Richland, WA 99352, USA; ^2^Center for Bioproducts and Bioenergy, Washington State University, Richland, WA, USA; ^3^Biological Sciences Division, Pacific Northwest National Laboratory, Richland, WA, USA; ^4^Computational Sciences and Mathematics Division, Pacific Northwest National Laboratory, Richland, WA, USA; ^5^Department of Neurobiology, 720 Westview Drive SW, Atlanta, GA, USA

## Abstract

The periplasm of Gram-negative bacteria is a dynamic and physiologically important subcellular compartment where the constant exposure to potential environmental insults amplifies the need for proper protein folding and modifications. Top-down proteomics analysis of the periplasmic fraction at the intact protein level provides unrestricted characterization and annotation of the periplasmic proteome, including the post-translational modifications (PTMs) on these proteins. Here, we used single-dimension ultra-high pressure liquid chromatography coupled with the Fourier transform mass spectrometry (FTMS) to investigate the intact periplasmic proteome of *Novosphingobium aromaticivorans*. Our top-down analysis provided the confident identification of 55 proteins in the periplasm and characterized their PTMs including signal peptide removal, N-terminal methionine excision, acetylation, glutathionylation, pyroglutamate, and disulfide bond formation. This study provides the first experimental evidence for the expression and periplasmic localization of many hypothetical and uncharacterized proteins and the first unrestrictive, large-scale data on PTMs in the bacterial periplasm.

## 1. Introduction 

The periplasm of Gram-negative bacteria is a hydrated gel located between the cytoplasmic and outer membranes and is comprised of peptidoglycan (cell wall), proteins, carbohydrates, and small solutes [1–3]. The periplasm is a dynamic subcellular compartment important for trafficking of molecules into and out of cells, maintaining cellular osmotic balance, envelope structure, responding to environmental cues and stresses, electron transport, xenobiotic metabolism, and protein folding and modification [4]. 

The periplasm provides a good model system to study protein biogenesis, composition, sorting, and modification at the molecular level. Indeed, it is analogous in many ways to the endoplasmic reticulum of eukaryotic cells in terms of transport, folding, and quality control [3]. Localization to the periplasm and beyond often involves an N-terminal secretion signal that targets the protein for translocation across the cytoplasmic membrane via the general secretory pathway [5]. These secretion signals (also known as signal peptides) are cleaved by signal peptidases located in the cytoplasmic membrane [6]. Thus, it is expected that signal peptide cleavage is a common modification in the periplasmic proteome. 

Compared to the cytoplasm, the periplasm is more vulnerable to changes in pH, temperature, and osmolarity in the external environment [4, 7, 8]. For structural stability in diverse and dynamic environmental conditions, periplasmic proteins often contain disulfide bonds and the periplasm is maintained in an oxidizing state to facilitate this process [9, 10]. Other PTMs, such as the addition of heme groups to cytochromes, may occur in the periplasm [11]. Therefore, the detailed study of bacterial periplasmic proteins not only allows for a better understanding of the physiology of microbial systems, but also provides information toward the complete annotation of mature proteoforms of microbial genomes, and it may give insight into protein sorting and PTMs in more complex systems.

The typical proteomic approach to profile the periplasm is the bottom-up liquid chromatography-tandem mass spectrometry (LC-MS/MS) approach [12, 13]. It has been applied to the periplasmic proteome of the extremophile *Acidithiobacillus ferrooxidans*, where it yielded a total of 131 proteins [14]. A majority of the identified proteins in *A. ferrooxidans* were categorized as periplasmic proteins based on their predicted export signals using software such as SignalP. However, direct evidence for signal peptide removal such as *N*-terminal peptide identifications was not available in that study. The most significant drawback of the bottom-up approach is that it rarely provides complete sequence coverage to ensure the identification of *N-*terminal peptides, which can be essential for understanding localization. Moreover, when multiple PTMs occur in a single protein, the bottom-up approach cannot accurately define proteoforms, as it does not have the ability to determine which combinations of PTMs cooccur in a single proteoform. 

To overcome these difficulties, we employ top-down mass spectrometry (MS) to study the periplasmic proteome. Top-down MS measures intact proteins and facilitates the full characterization of proteoforms including PTMs [15]. The top-down approach has been successfully applied for the characterization of various protein PTMs including signal peptide identification [16]. Recent improvements in intact protein LC separations and high performance FTMS instrumentation greatly expand the observable range of proteoforms. Because top-down analysis preserves the mature N-terminus, the proteolytic processing (e.g., N-terminal cleavage) of a protein is evident. Thus top-down MS provides an experimental validation of bioinformatic predictions such as the signal peptide cleavage predicted by SignalP. 

As an initial subject for analysis, we focused on the Gram-negative alphaproteobacterium, *Novosphingobium aromaticivorans*. Members of this genus are noted for their remarkable ability to degrade a variety of aromatic hydrocarbons [17]. The genome of only one species, *N. aromaticivorans,* has been completely sequenced. In a genome with 3917 proteins, nearly 30% are annotated as “hypothetical”; moreover, using the subcellular localization predictor PSORTb (http://www.psort.org/psortb/), 33% of proteins have “unknown” localization. Our current goal is to identify protein constituents of the periplasm of this unusual microorganism, to aid in annotation of hypothetical and poorly characterized proteins, and to survey, in an unrestricted manner, the PTMs existing in these proteins. We here report our results on profiling the enriched periplasmic proteome of *N. aromaticivorans* using a high throughput intact protein (top-down) analysis. A total of 55 proteins were confidently identified, and their PTMs were characterized including N-terminal processing (e.g., signal peptide removal), acetylation, glutathionylation, pyroglutamate modification, and disulfide bond formation. 

## 2. Experimental Section

### 2.1. Periplasmic Protein Extraction

*N. aromaticivorans* str. DSM 12444 was grown to early stationary phase aerobically in 50% (v/v) Luria Bertani broth. Cells were harvested by centrifugation at 8,500 g for 5 min, washed once with sodium phosphate (pH 7.5), and the periplasm extracted as previously reported [18]. The soluble periplasmic fraction was flash-frozen in liquid nitrogen and concentrated using a SpeedVac (Thermo-Savant) prior to top-down analysis. For peptide-level analysis, 4 volumes of 20% acetonitrile and 3 volumes of water were added, followed by trypsin (trypsin to protein ratio 1 : 50), and incubated at 37°C for 18 h. The sample was concentrated to dryness in a SpeedVac and suspended in 20 *μ*L 0.1% formic acid prior to LC-MS/MS analysis.

### 2.2. Intact Protein LC-MS/MS Analysis

The intact protein RPLC separation was performed on a Waters NanoAcquity system with a column (80 cm × 75 *μ*m i.d.) packed in-house with Phenomenex Jupiter particles (C5 stationary phase, 5 *μ*m particle size, 300 Å pore size). Mobile phase A was composed of 0.5% acetic acid, 0.01% TFA, 5% isopropanol, 10% acetonitrile (ACN), and 84.5% water. Mobile phase B consisted of 0.5% acetic acid, 0.01% TFA, 9.9% water, 45% isopropanol, and 45% ACN. The operating flow rate was 0.3 *μ*L/min. The RPLC system was equilibrated with 100% mobile phase A for 5 minutes and then increased to 20% mobile phase B in 1 minute. A 250 minute linear gradient was set from 20% mobile phase B to 55% mobile phase B. MS analysis was performed using an LTQ-Orbitrap Velos spectrometer (Thermo Scientific, San Jose, CA) outfitted with a custom electrospray ionization (ESI) interface. ESI emitters were custom made using 150 um o.d. × 20 um i.d. chemically etched fused silica [19]. The heated capillary temperature and spray voltage were 275°C and 2.2 kV, respectively. Two LC-MS/MS analyses were performed: one with ETD fragmentation and one with HCD fragmentation. For the LC-MS/MS analysis with ETD fragmentation, a parent spectrum was collected at a 60 K resolution and was followed by high resolution (30 K) ETD MS/MS of the 8 most intense ions from the parent spectrum. The ETD reaction time was fixed at 40 ms. For the LC-MS/MS analysis with HCD fragmentation, a parent spectrum was collected at a 60 K resolution and was followed by high resolution (30 K) HCD MS/MS of the 8 most intense ions from the parent spectrum. FT MS/MS employed 45% normalized collision energy for HCD. Mass calibration was performed prior to analysis according to the method recommended by the instrument manufacturer.

### 2.3. Capillary LC-MS/MS Analysis on Trypsin-Digested Peptides

Bottom-up identification of proteins was achieved through the detection of peptides with LC-MS/MS. The capillary RPLC system used for peptide separations has been previously described [20]. Briefly, the HPLC system consisted of a custom configuration of 100 mL ISCO Model 100DM syringe pumps (Isco, Inc., Lincoln, NE), 2-position Valco valves (Valco Instruments Co., Houston, TX), and a PAL autosampler (Leap Technologies, Carrboro, NC), allowing for fully automated sample analysis across four separate HPLC columns (3 *μ*m Jupiter C18 stationary phase, Phenomenex, Torrance, CA). Mobile phases consisted of 0.1% formic acid in water (A) and 0.1% formic acid acetonitrile (B). The HPLC system was equilibrated at 10 kpsi with 100% mobile phase A, and a mobile phase selection valve was switched 50 min after injection, which created a near-exponential gradient as mobile phase B displaced A in a 2.5 mL active mixer. A 40 cm length of 360 *μ*m o.d. × 15 *μ*m i.d. fused silica tubing was used to split ~17 *μ*L/min of flow before it reached the injection valve (5 *μ*L sample loop). The split flow controlled the gradient speed under conditions of constant pressure operation (10 kpsi). Flow through the capillary HPLC column when equilibrated to 100% mobile phase A was ~500 nL/min. ESI using an etched fused-silica tip [19] was employed to interface the RPLC separation to an LTQ mass spectrometer (Thermo Scientific, San Jose, CA). Precursor ion mass spectra (automatic gain control was set to 1 × 10^6^) were collected for 400–2000 m/z range at a resolution of 100 K followed by data dependent ion trap CID MS/MS (collision energy 35%, AGC 3 × 10^4^) of the ten most abundant ions. A dynamic exclusion time of 180 sec was used to discriminate against previously analyzed ions.

### 2.4. Data Analysis

Intact protein MS/MS data were subjected to data analysis and protein identification using MS-Align+^16^ (http://bix.ucsd.edu/projects/msalign/) with the following search parameters: minimal precursor mass = 2500 Da; minimal fragment peaks per scan = 10; maximum number of modifications = 2; fragment mass error tolerance = 15 ppm. MS-Align+ reported only the PrSM with the best E-value for each spectrum. LC-MS/MS data were searched against the Genbank protein annotation (accession CP000248). The false discovery rate (FDR) for protein/spectrum matches was estimated by searching all top-down spectra against the human Uniprot database. A final cutoff of E-value 2.7E^−4^ was used to achieve FDR 1%. Protein identifications were further manually verified. Peptide-level MS/MS data were searched using SEQUEST and were filtered using MSGF [21] with a spectral probability cutoff of 1E^−10^. All the raw datasets and MSAlign+ output results were deposited at http://omics.pnl.gov/view/publication_1074.html.

Signal peptides were determined using the identified peptides and the prokaryotic proteogenomic pipeline [22]. The three criteria were taken from previously recognized signal peptide characteristics [23]. We required a hydrophobic patch of at least eight contiguous amino acids and examined the signal peptide C-terminus for the expected A-X-B cleavage motif (where A = [Ile, Val, Leu, Ala, Gly, Ser, Thr], X = any amino acid, B = [Ala, Gly, Ser]). We also required a basic residue between the start and the hydrophobic patch. Typically the mature protein starts between 15 and 35 residues from the initiator methionine. However, due to the possibility of incorrectly annotated start sites, we allowed for some variance from this requirement. 

Subcellular localization and protein functional predictions were made using PSORTb (http://db.psort.org/browse/genome?id=8602), SignalP (http://www.cbs.dtu.dk/services/SignalP/), SecretomeP (http://www.cbs.dtu.dk/services/SecretomeP/), and the Comprehensive Microbial Resource Genome Tools (http://cmr.jcvi.org/tigr-scripts/CMR/shared/Genomes.cgi).

## 3. Results and Discussion

**Figure 1:**
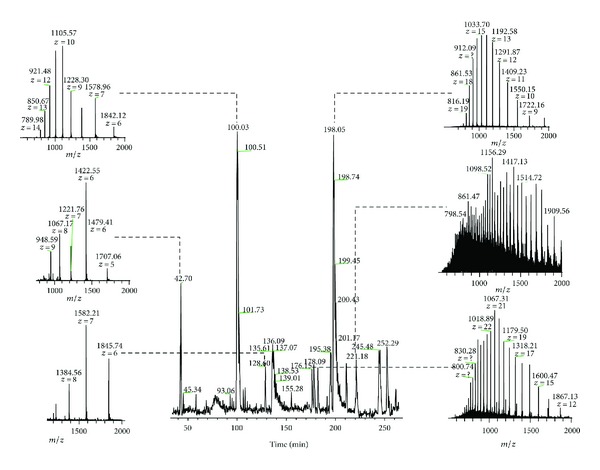
Total ion chromatogram (TIC) of an RPLC-MS analysis of intact periplasmic protein from *N. aromaticivorans. *Several representative intact protein spectra are highlighted.

To study the mature proteoforms and PTMs of the *N. aromaticivorans* periplasm, the periplasmic fraction was prepared as previously reported [18]. The enriched intact periplasmic protein fraction was subjected to nano-LC-MS/MS using two different fragmentation methods.  Figure 1 shows the base peak chromatogram of a 300-minute LC-MS analysis with several representative intact protein spectra. The detected protein masses varied from 4 kDa to 40 kDa. Intact MS/MS data were analyzed using MS-Align+ [15]. In total, 55 proteins were identified at a 1% FDR (Table 1). 

**Figure 2:**
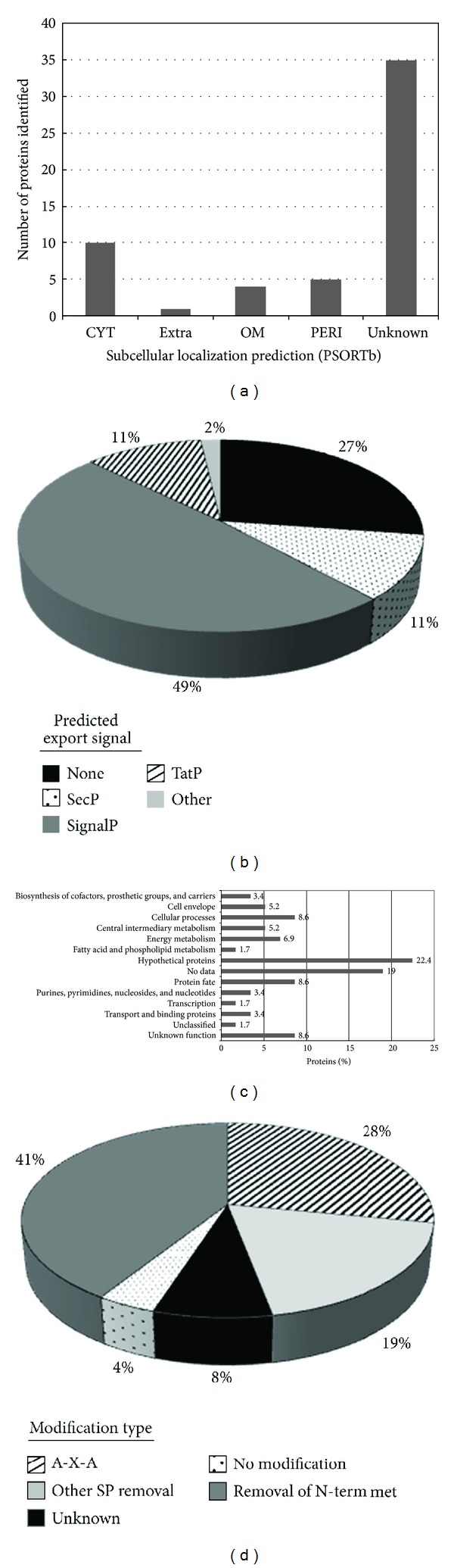
(a) Subcellular localization prediction using PSORTb: cytoplasmic (CYT), inner membrane (IM), extracellular (EXTRA), outer membrane (OM), and periplasmic (PERI). (b) Pie chart showing the distribution of predicted export signals among proteins identified by top-down MS. One predicted PERI protein with no detected export signal is designated as “Other.” (c) JCVI annotated functional categories of intact proteins identified using top-down analysis. (d) Pie chart representation of protein modifications observed by top-down MS.

To highlight the specificity and efficiency of the enrichment, we note that abundant cytoplasmic proteins were largely absent in the periplasmic preparation, indicating a low amount of cell lysis during the experiments. For example, none of the ribosomal proteins were detected. Small, highly abundant cytoplasmic proteins such as ribosomal proteins typically dominate in global (whole cell) top-down LC-MS analyses and are often detected in membrane fractions [24, 25]. Several of the proteins identified here were expected to be localized to the periplasm. For example, superoxide dismutase (Saro_0483), a tetratricopeptide repeat protein (Saro_0209), and two peptidyl-prolyl cis-trans isomerases (Saro_0837 and Saro_2251) are known to be localized in the periplasm in other Gram-negative bacteria. Moreover, these proteins were predicted by PSORTb to be periplasmic in *N. aromaticivorans*, and the latter three were enriched in the periplasm of this organism, compared to the cytoplasm, inner membrane, or outer membrane fractions, in a proteomic analysis of multiple subcellular fractions (data not shown). Also, it should be noted that some outer membrane proteins have periplasmic domains. For example, half of OmpA (residues 172–325) is periplasmic [26], resulting in its identification in the periplasm here. Subcellular localization predictions of the identified proteins are shown in Figure 2(a). The majority fall into the “unknown” category, making this the first experimental data on subcellular localization for these proteins. 

The first and most prevalent type of PTM identified via the top-down approach was proteolytic cleavage. The cleavage events described later were found to be uniformly present; there were no uncleaved forms of the protein detected. We categorized identified proteoforms according to known types of proteolytic maturation. Based on the observed signal peptide cleavage, 25 proteins were localized to the periplasm via Sec-dependent secretion (Figure 2(b)) with detected signal peptide removal. Upstream of the mature protein, the three hallmarks of signal peptides were clearly present: early basic residue(s), a hydrophobic patch of at least 8 residues, and the signal peptidase I cleavage motif. Sixteen proteins were detected with the predominant Ala-Xxx-Ala motif, while 7 of them exhibited tolerated variability at the −3 position [23]. Many of these proteins had poor functional characterization, with 21 lacking any functional annotation (Figure 2(c)) or significant match to protein domain descriptors (e.g., CDD or Pfam; Table 1). Thus, by identifying both their cellular location (periplasm) and their maturation processing, we have significantly added to the annotation of these proteins. We compared the observed signal peptide cleavage to the computational predictions from SignalP4.0 (Figure 2(b)). SignalP correctly predicted 23 of the 25 proteins as containing a signal peptide but did not determine the correct site of cleavage in 6 of the 23 (Table 1) based on top-down analysis. Moreover, SignalP had two false predictions where the identified proteoform lacked a cleaved signal peptide. Other computational tools were also applied such as TatP and SecP, yielding six more proteins with predicted export signals. Among these six proteins, only two were confirmed with cleaved signal peptides through top-down analysis. Therefore, top-down analysis provided additional information for confident protein categorization, which can be potentially incorporated with currently available software tools to further improve the prediction performance.

We observed that almost all the proteins not exhibiting signal peptide removal had methionine excision (Figure 2(d) and Table 1). Of the 26 proteins that did not show signal peptide removal or other large N-terminal cleavages, 24 of them began at the second amino acid. The penultimate residue was always consistent with N-terminal methionine excision (NME): alanine, proline, threonine, serine, or glycine [27]. Given the background amino acid frequency and the expected efficiency of methionine amino peptidase [28], the binomial probability of observing such a concentration of NME matured proteins in the periplasm is 4.7 E-6. For comparison, a global top-down analysis of *E. coli* done recently in our lab produced a 1 : 1 ratio, 69 proteins without methionine excision, and 70 proteins with methionine excision (unpublished results). Additionally, a proteomic and bioinformatic analysis of NME revealed that only a minority of the proteins in a given proteome are subject to NME [29]. The functional significance for pervasive NME in the periplasm is not clear but may be related to protein stability in the potentially hazardous periplasmic environment.

**Figure 3:**
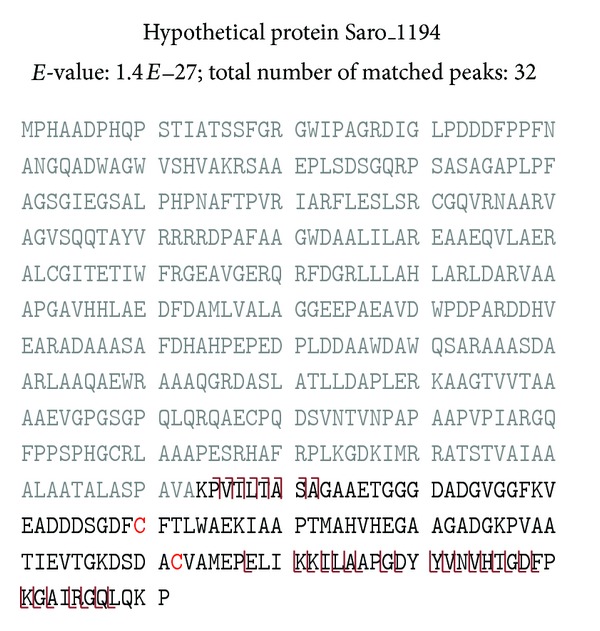
Fragmentation ion map of the uncharacterized protein Saro_1194 using intact protein MS/MS, indicating that the mature protein contains only the C-terminal portion of the predicted protein sequences starting at residue 414 (the portion of the sequence labeled with grey font was not detected in this experiment).

Some of the identified proteins displayed large N-terminal cleavages. For example, the uncharacterized protein Saro_1194 was observed in the data as a mature protein containing only the extreme C-terminal portion of the protein sequence, starting at residue 414, immediately after A-V-A (Figure 3). BLAST analysis showed that the annotated sequence always matched to two separate proteins, well demarcated at the N-terminal and C-terminal extremes of the protein (Figure S1). The C-terminal portion, which was identified from the top-down MS data, also exhibited partial homology to the CHRD domain (pfam07452). Additionally, in two closely related *Erythrobacter* species, the two BLAST hits form a syntenic block in the genome. It is not uncommon for bacteria to combine proteins into multidomain or multifunctional proteins. However, the finding of the mature protein with a perfectly matched and cleaved signal peptide (upstream of the A-V-A is an easily detectable hydrophobic patch and basic residues) suggests that Saro_1194 is actually two separate proteins. 

 The bacterial periplasm is an oxidizing environment that facilitates disulfide bond formation for correct protein folding and stability [9, 10]. Fifty-three of the 55 proteins identified using our top-down approach contained an even number of cysteines within the detected sequences (i.e., after removal of the signal peptides), including 33 proteins containing no cysteine, 17 proteins containing two cysteines, one protein containing four cysteines, and two proteins containing eight cysteines. Two proteins contain a single cysteine, phosphoribosylformylglycinamidine synthetase PurS, and the uncharacterized protein PhnA. Among the proteins containing two cysteines, only two proteins (arsenate reductase and molybdopterin binding domain, Table 1) did not form a disulfide bond. Instead, both of these proteins contained a glutathionylated adduct (RSSG) on one of the cysteine residues. Although neither of these proteins was detected with signal peptide removal, it has been reported that under certain conditions, 90% of the arsenate reductase activity was found in the periplasmic faction in some bacteria (e.g., *Shewanella* [30]). In other bacteria, several molybdopterin binding proteins (e.g., periplasmic nitrate reductase from *Desulfovibrio desulfuricans* ATCC 27774) were also found in periplasmic fractions [31, 32]. Therefore, these two proteins are likely to be periplasmic proteins, and the observation may indicate the occurrence of cysteine glutathionylation as a form of oxidation in the periplasm other than disulfide bond based oxidation. 

Proteoform identifications from top-down also found other PTMs (Table 1). The most common was pyroglutamate, which was very often found on signal peptide cleaved proteins. In nine proteins where the first residue of the mature protein was glutamine, a conversion to pyroglutamate was observed. As mentioned earlier, two proteins were observed with S-glutathiolation, Saro_1778 molybdopterin binding domain protein and Saro_3279 arsenate reductase.

**Figure 4:**
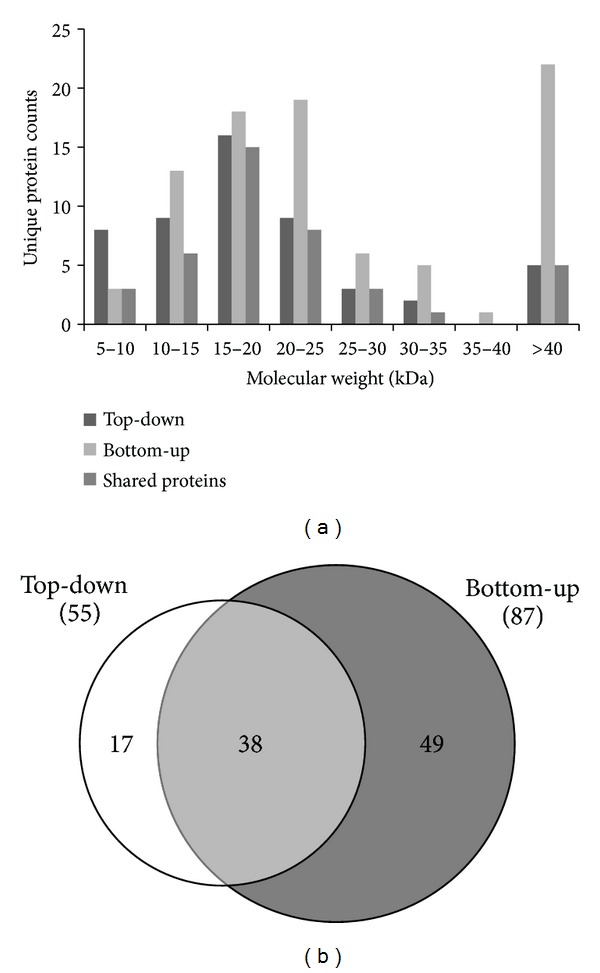
(a) Molecular mass distributions of proteins identified using top-down and bottom-up analysis. Theoretical molecular masses were calculated using amino acid sequence. (b) Overlap of proteins identified using top-down and bottom-up analysis (considering proteins identified by at least two unique peptides).

To access the sensitivity and depth of the top-down approach, the same periplasmic enriched protein fraction was analyzed by bottom-up proteomics (Supplementary Table 1 available online at http://dx.doi.org/10.1155/2013/279590). In the bottom-up analysis, 87 proteins were confidently identified with at least two unique peptides. Of these proteins, 37 were also identified in the top-down approach. Fifty proteins were detected only in the bottom-up approach, but most of them have molecular masses larger than 40 kDa (Figure 4(a)), which makes them less amenable to top-down analysis at present. Seventeen proteins were uniquely identified in the top-down approach (Figure 4(b)); eight of these proteins have molecular masses less than 10 kDa. Characterizing small proteins represents a challenge for the bottom-up workflow due to the inability to generate sufficient tryptic peptides for analysis. 

**Figure 5:**
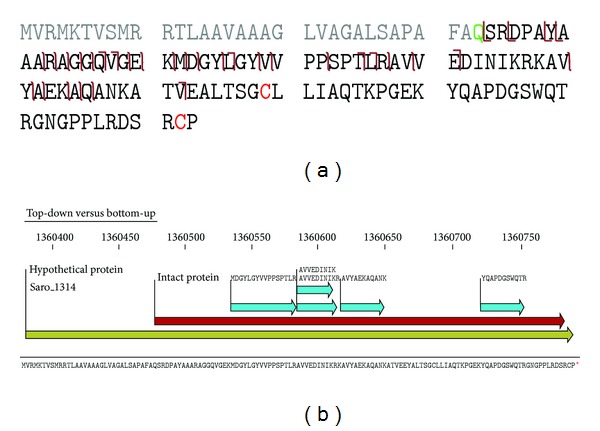
Top-down and bottom-up analysis of the hypothetical protein Saro_1314. (a) Fragmentation ion map illustrating high confidence identifications (“Q” highlighted in green font was modified as pyroglutamic acid, and two “C” highlighted in red font formed a disulfide bond). (b) Sequence coverage between top-down approach and bottom-up approach (blue arrows indicate the sequences identified using bottom-up approach).

We compared the top-down and bottom-up data for their ability to detect mature protein isoforms. The bottom-up data identified 14 proteins with signal peptide cleavage, of which seven were also identified by top-down analysis. Of the remaining seven that were unique to bottom-up, four were large proteins (>45 kDa) and thus largely inaccessible using our current top-down MS platform. We note that for 12 additional proteins, the signal peptide was identified only in the top-down approach, while peptides found in the bottom-up data did not identify a signal peptide cleavage (i.e., none of the peptides captured the mature N-terminus). For example, a hypothetical protein Saro_1314 was confidently identified with a signal peptide removal, a disulfide bond between Cys 99 and Cys 132, and an N-terminal pyroglutamate modification (Figure 5(a)). Only five tryptic peptides were detected for the same protein using the bottom-up approach, and none of them provided evidence for the PTMs (Figure 5(b)). Thus, while the bottom-up approach led to the identification of a larger number of proteins (i.e., a larger survey of periplasmic contents), the top-down analysis provided information on the mature N-terminus and other PTMs. 

## 4. Conclusions

Top-down MS analysis of the intact periplasmic fraction of *N. aromaticivorans* indicated the extensive use of sec-dependent signal peptides and disulfide bond formation, as expected for a Gram-negative periplasm. Less expected was the high frequency of NME, which, to our knowledge, has not previously been reported in the bacterial periplasm. Considering these two forms of cleavage and protein maturation, almost all the proteins detected in this study were modified. Moreover, these are cleavage maturation events where no evidence was found of the unmodified protein. Although various modification types were detected, the predominant PTM observed here was proteolysis. Beyond simply showing expression of several “hypothetical” proteins, we have improved the annotation of many genes by providing localization and PTM status, which provides a basis for further functional annotation of this poorly characterized genus. We propose that top-down MS should be an integral part of efforts towards the characterization of bacterial proteomes in the future.

## Supplementary Material

Supplemental Table 1: provides an annotated list of proteins confidently identified in the enriched periplasmic fraction by bottom-up LC-MS/MS.

## Figures and Tables

**Table 1:** Modifications of identified proteins using top-down approach.

Locus_Tag	Genbank Protein Desc	Export signal	Detected signal peptide^a^	N-terminal	Other modifications
Saro_2004	Alkyl hydroperoxide reductase/Thiol specific antioxidant/Mal allergen	SecP	None	Removal of met	
Saro_2586	Cold-shock DNA-binding protein family	SecP	None	Removal of met	
Saro_0565	Glutathione peroxidase	SecP	None	Removal of met	
Saro_0483	Superoxide dismutase	SecP	None	Removal of met	
Saro_3290	Thiamine biosynthesis protein ThiS	SecP	None	Removal of met	
Saro_1996	Thioredoxin	SecP	None	Removal of met	Disulfide bond
Saro_1332	CsbD-like protein	SecP/TatP	None	Removal of met	
Saro_1919	Hypothetical protein	SecP/TatP	Unknown	Proteolytic fragment	
Saro_1314	Conserved hypothetical protein	SecP/TatP	Yes	AXA	Disulfide bond, pyro glu
Saro_2385	Hypothetical protein	SecP/TatP	Yes	AXA	Pyro glu
Saro_3257	Conserved hypothetical protein	SignalP	None	Proteolytic fragment	
Saro_3518	Cupin 2, conserved barrel domain protein	SignalP	None	Removal of met (wrong starting site)	
Saro_3173	OmpA/MotB	SignalP	None	Proteolytic fragment	
Saro_1303	Hypothetical protein	SignalP	Unknown	Proteolytic fragment	
Saro_1685	Amine dehydrogenase	SignalP	Yes	AXA	
Saro_2852	Ankyrin	SignalP	Yes	AXA	Pyro glu
Saro_3053	Beta-Ig-H3/fasciclin	SignalP	Yes	AXA	
Saro_0830	Cell wall surface anchor family protein	SignalP	Yes	VAA, not AXA	
Saro_2955	Conserved hypothetical protein	SignalP	Yes	AXA	
Saro_1378	Conserved hypothetical protein	SignalP	Yes	AXA	
Saro_0103	Conserved hypothetical protein	SignalP	Yes	AXA	Disulfide bond
Saro_2067	Conserved hypothetical protein	SignalP	Yes	SHA, not AXA	Pyro glu
Saro_1721	Conserved hypothetical protein	SignalP	Yes	THA, not AXA	
Saro_2522	Hypothetical protein	SignalP	Yes	ASN, not AXA	Disulfide bond
Saro_3326	Hypothetical protein	SignalP	Yes	AXA	Disulfide bond
Saro_2384	Hypothetical protein	SignalP	Yes	AXA	Pyro glu
Saro_1978	Hypothetical protein	SignalP	Yes	AXA	Pyro glu
Saro_1502	Hypothetical protein	SignalP	Yes	AXA	
Saro_1412	Hypothetical protein	SignalP	Yes	AXA	Disulfide bond
Saro_2350	Peptidase M28	SignalP	Yes	AXA	
Saro_0837	Peptidyl-prolyl cis-trans isomerase, cyclophilin type	SignalP	Yes	LVA, not AXA	
Saro_2251	Peptidylprolyl isomerase	SignalP	Yes	VAA, not AXA	Pyro glu
Saro_0989	Peptidylprolyl isomerase, FKBP-type	SignalP	Yes	AIS, not AXA	Disulfide bond
Saro_0823	Protein of unknown function DUF192	SignalP	Yes	AXA	
Saro_3075	TonB-dependent receptor	SignalP	Yes	AXA	Proteolytic fragment
Saro_2265	YceI	SignalP	Yes	MVA, not AXA	Pyro glu
Saro_1171	Hypothetical protein	SignalP/SecP	None	Removal of met	Disulfide bond
Saro_1420	Antibiotic biosynthesis monooxygenase	TatP	None	Removal of met	Disulfide bond
Saro_3279	Arsenate reductase	TatP	None	N/A	S-glutathiolation on cysteine
Saro_1703	(2Fe-2S)-binding protein		None	Removal of met	Disulfide bond
Saro_1346	(2Fe-2S)-binding protein		None	Removal of met	Disulfide bond
Saro_1339	Acyl carrier protein		None	Removal of met	Modification (382 Da)
Saro_2520	BolA-like protein		None	Removal of met	
Saro_0034	Chaperonin Cpn10		None	Removal of met	
Saro_2299	Conserved hypothetical protein		None	Removal of met	
Saro_2229	GreA/GreB family elongation factor		None	Removal of met	Both acetylation and n/a
Saro_2403	H+-transporting two-sector ATPase, delta/epsilon subunit		None	Removal of met	
Saro_1768	Hypothetical protein		None	Removal of met	
Saro_1177	Hypothetical protein		None	Removal of met	
Saro_1778	Molybdopterin binding domain		None	Removal of met	S-glutathiolation on cysteine
Saro_0894	Nucleoside diphosphate kinase		None	Removal of met	
Saro_1830	PhnA protein		None	Removal of met	
Saro_1033	Phosphoribosylformylglycinamidine synthetase PurS		None	N/A	
Saro_0209	Tetratricopeptide TPR_4		None	Removal of met (wrong starting site)	Pyro glu
Saro_1194	Hypothetical protein		Yes	AXA, unusual^b^	Disulfide bond

^a^Signal peptide cleavage was annotated as described in the Experimental section.

^b^The unusual cleavage site was further validated. See discussion in text and Figure 3.
